# Sudden loss of ventilation through a double lumen endotracheal tube requiring emergency provision of a surgical airway by bronchotomy

**DOI:** 10.1186/1749-8090-8-S1-P138

**Published:** 2013-09-11

**Authors:** Robert Torrance, Alan Dawson, Jared M Wohlgemut, Keith Buchan

**Affiliations:** 1 School of Medicine and Dentistry, University of Aberdeen, Aberdeen, UK; 2 Department of Cardiothoracic Surgery, Aberdeen Royal Infirmary, Aberdeen, UK

## 

A left completion pneumonectomy operation for primary lung cancer (left lower lobectomy) was complicated by sudden loss of ability to ventilate the patient via the double lumen endotracheal tube. The problem could not be overcome by the anesthesiologist. In the face of impending cardiorespiratory arrest a single lumen tube was introduced through an incision in the left main bronchus through to the right main bronchus. This life-saving manoeuvre safeguarded the airway and permitted a successful outcome to the operation.

